# Microfluidic Sorting of Cells by Viability Based on Differences in Cell Stiffness

**DOI:** 10.1038/s41598-017-01807-z

**Published:** 2017-05-17

**Authors:** Muhymin Islam, Hannah Brink, Syndey Blanche, Caleb DiPrete, Tom Bongiorno, Nicholas Stone, Anna Liu, Anisha Philip, Gonghao Wang, Wilbur Lam, Alexander Alexeev, Edmund K. Waller, Todd Sulchek

**Affiliations:** 1 George W. Woodruff School of Mechanical Engineering, Georgia Institute of Technology, 801 Ferst Drive, Atlanta, GA 30332-0405 USA; 2 Wallace H. Coulter Department of Biomedical Engineering, Georgia Institute of Technology, 313 Ferst Drive, Atlanta, GA 30332-0535 USA; 3 Department of Pediatrics, Division of Pediatric Hematology/Oncology, Aflac Cancer Center and Blood Disorders Service of Children’s Healthcare of Atlanta, Emory University School of Medicine, Atlanta, Georgia 30322 USA; 4 Winship Cancer Institute, Emory School of Medicine, 1365 Clifton NE Rd, Atlanta, GA 30322 USA

## Abstract

The enrichment of viable cells is an essential step to obtain effective products for cell therapy. While procedures exist to characterize the viability of cells, most methods to exclude nonviable cells require the use of density gradient centrifugation or antibody-based cell sorting with molecular labels of cell viability. We report a label-free microfluidic technique to separate live and dead cells that exploits differences in cellular stiffness. The device uses a channel with repeated ridges that are diagonal with respect to the direction of cell flow. Stiff nonviable cells directed through the channel are compressed and translated orthogonally to the channel length, while soft live cells follow hydrodynamic flow. As a proof of concept, Jurkat cells are enriched to high purity of viable cells by a factor of 185-fold. Cell stiffness was validated as a sorting parameter as nonviable cells were substantially stiffer than live cells. To highlight the utility for hematopoietic stem cell transplantation, frozen samples of cord blood were thawed and the purity of viable nucleated cells was increased from 65% to over 94% with a recovery of 73% of the viable cells. Thus, the microfluidic stiffness sorting can simply and efficiently obtain highly pure populations of viable cells.

## Introduction

The presence of nonviable cells in cell suspension causes a ubiquitous problem in the biomedical field. Typical cell cultures can present with 5–20% of nonviable cells^1–4^, which may confound the accuracy of any assay measuring cellular product or function. In some sensitive cell cultures, such as of embryonic stem cells, the presence of necrotic cells release factors that negatively impact the health of the entire culture in a cascading manner^2, 5^, therefore periodic removal of dead cells improve﻿s﻿ culture health. Also the presence of nonviable cells can be detrimental to clinical outcomes of cell therapies, for example the delaying of engraftment time of hematopoietic stem cell transplantation^6–8^. While the immune system of organisms can identify and remove dead and dying cells from the body, the presence of debris and dead cells in *in vitro* cell culture adversely affects its quality and productivity. Enrichment for viable cells can also improve the accuracy of biomedical assays, as quantification can be made inaccurate by the presence of nonviable cells in sample preparation or lead to improper conclusions in monitoring the effect of experimental compounds^9–12^.

One additional importance of live/dead cell sorting is obtaining high purity of cell-based therapies after the cell manufacturing and storage process. Cellular biomanufacturing requires large-scale, high-quality cells with consistency and reproducibility^13, 14^. Manufacturers should meet release criteria for the number and percentage of viable cells, which if not satisfied leads to unpredictability of the treatment. For example a viability of 85% or greater is recommended by the FDA for transplantation of cord blood cells^15–17^. Cellular products should also be tested for potency, which is made more inconsistent with the presence of variable numbers of nonviable cells.

In adherent cell culture, nonviable cells can be removed by basis of their lack of attachment to the tissue culture flask, though this is not an option for many cell cultures and manufacturing processes. A number of labeled cell sorting approaches are sensitive to viability, for example flow cytometry with propidium iodide staining^18, 19^ and monoclonal antibody-magnetic bead pull down assay^20^, however, these methods require modifying the cells with expensive reagents and time consuming processing^21^. Consequently, label-free techniques that are high throughput and accurate are needed as an alternative.

The use of biophysical differences between live and dead cells could greatly benefit viability separation technologies. Biophysical properties of cells have been effectively used for sorting and enhanced detection of numerous diseases, including cancer, malaria, and sickle cell anemia^22–26^. In regards to cell viability, biophysical differences between live and dead cells have been identified, including size^27, 28^, density^28^, stiffness^27, 29^, viscosity^30^, weight^31^, and electrical polarizability^9, 10, 32, 33^. These properties can be exploited for the label-free sorting and isolation of viable cells^10, 11^. Density gradient centrifugation can also remove dead cells from a population, but the bulk processing approach suffers from a significant loss of live cells due to overlapping properties of size and density^28^.

**Figure 1:**
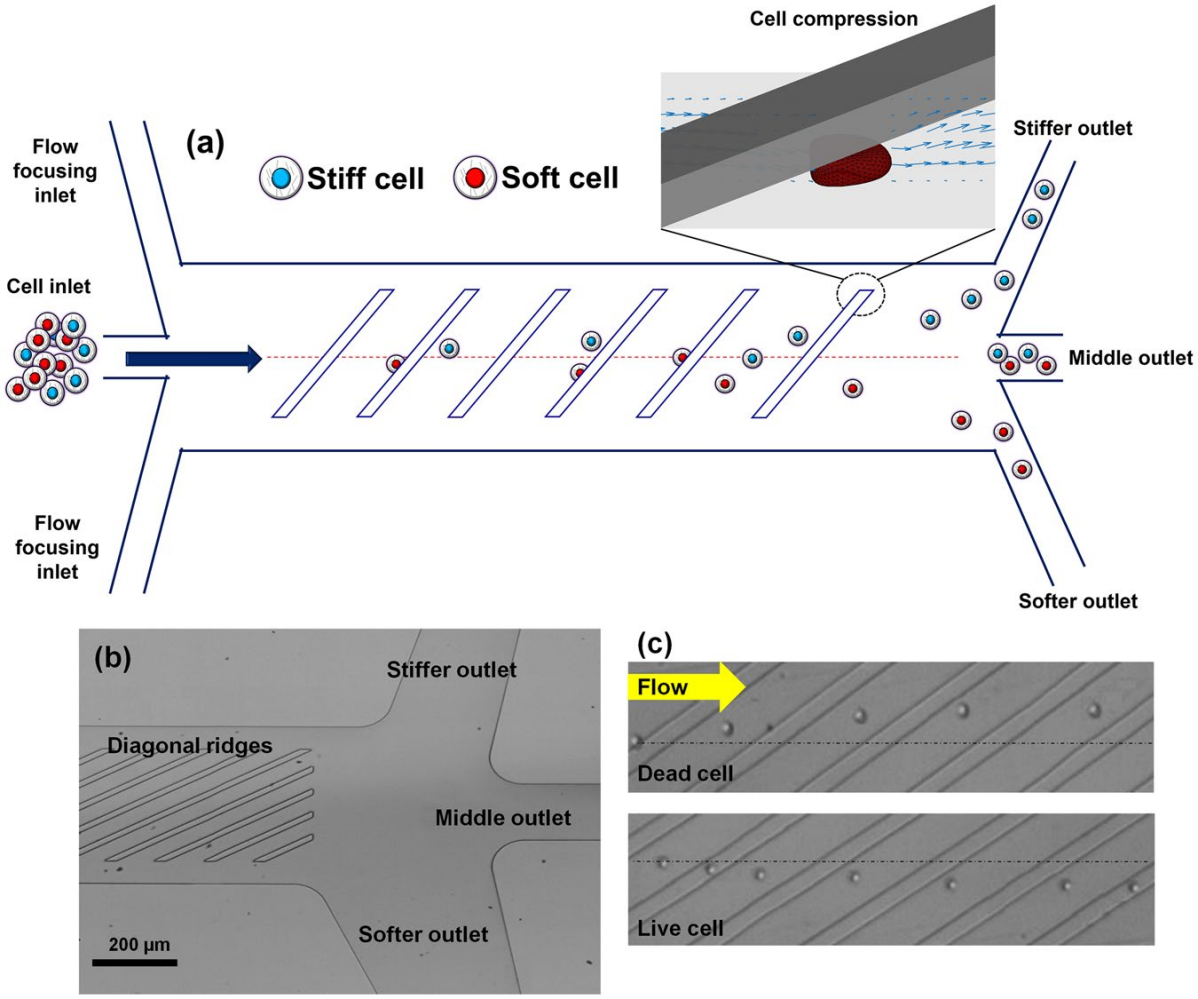
Ridge based microfluidic device for viable cell sorting. (**a**) Schematic representation of the device showing that cells will squeeze through ridges and flow towards different outlets based on their stiffness differences; (**b**) optical micrograph of the fabricated device showing the ridges and outlets; (**c**) representative micrograph of overlaid video of dead and live cells moving towards opposite directions in the device. The yellow arrow show the direction of fluid flow﻿.

Microfluidic platforms have also been used to separate live and dead cells through differences in electrical (i.e., conductivity and permittivity) and mechanical (i.e., size and shape) properties of cells^9, 10, 33, 34^. Various modes of dielectrophoresis using alternating current^35^, hydrodynamic^36^, insulator-based^37^, and contactless^11^ approaches have been reported to sort viable cells. These approaches can be limited by throughput or may not offer a sufficient balancing between sensitivity of dead cell removal while maintaining a high live cell recovery^9, 35, 37^. Thus alternate high-throughput approaches are needed for continuous sorting of live and dead cells with an active ability to provide higher purity and recovery of viable cells. We have developed a simple and label-free microfluidic device to sort live and dead cells by differences in their stiffness. The device consists of periodic diagonal ridges oriented skew to the direction of fluid flow, as shown in Fig. 1. Flowing cells are compressed by the ridge constrictions and translated perpendicular to the channel axis in relation to cellular biomechanical properties, with contributions from elastic forces of cell deformation as well as hydrodynamic forces resulting from helical flow patterns inside the channel^24^. The balance of these forces results in stiff cells being directed to upper outlets and softer cells to lower outlets as illustrated in Fig. 1(a).

Jurkat cells were used in the proof of principle experiments reported. Heat treatment was applied to Jurkat cells to initiate cell death^29, 34^, which was confirmed using ethidium homodimer-1 (EthD-1) staining and flow cytometry. Atomic force microscopy measurements were conducted to quantify the increase in cell stiffness and dead cells were found to be more than three times stiffer than live cells, consistent with previous studies^27, 29, 38, 39^. Cells were injected through a focused cell inlet and multiple outlets in the device provided a capability to select for greater viable cell purity and greater viable cell collection efficiency. As a practical demonstration of device capabilities, the device was also used to increase the viability of thawed cord blood samples, which could have future applications in improving the efficacy of hematopoietic stem cell transplantation. The ridged channel design has previously been demonstrated as an effective means for cell sorting by stiffness, size, and viscosity^22–24, 40–42^, and this study is the first to demonstrate that stiffness differences can be used for viability sorting, as illustrated in the unique trajectories of live and dead cells observed in Fig. 1(c).

## Materials and Methods

### Fabrication of Microfluidic Device

The microfluidic device was fabricated using replica molding of polydimethylsiloxane (PDMS) on a SU-8 patterned silicon wafer^22–24^. All devices tested were designed in *AutoCAD* and flow trajectories were simulated in ANSYS Fluent. The molds for the device were fabricated on a silicon wafer by spin coating SU-8 2007 (SU-8 2007, Microchem Corp.) using a two-layer photolithography process. The dimensions of the molds, particularly the ridge heights, were measured with profilometry (Dektak 150 profiler) and optical microscopy. Several device parameters influence cell trajectories, which include ridge gap distance, number of ridges, and angle of ridges. These effects were thoroughly studied in previous publications^22–24^. Based on the previous studies the ridge angle, total number of ridges, and ridge gap were chosen to be 30 °, 17, and 9 µm, respectively. The ridge gap was chosen to be small enough to compress the cells sufficiently without clogging the device and compares to an average cell diameter of 15.3 µm^22, 24, 43^. Three and five outlet devices were designed to understand how the ability to fractionate the output can improve cell purity^22, 24, 43^. Also, the number of ridges was optimized to 14 for 5-outlets device based upon empirical data obtained from 3-outlets device which showed that the trajectory divergence cease as cells lose their effective compliance^22, 24^. In addition, the diameter of the outlets was increased to 3 mm for increased cell collection. The mold pattern was transferred to polydimethylsiloxane (PDMS), mixed (10:1, wt/wt) with Sylgard 184 silicone elastomer curing agent (Dow Corning). This mixture was degassed in a vacuum chamber, poured on the mold and cured at 60 °C temperature for several hours. Then inlet and outlet holes were punched with a biopsy punch. The PDMS device was bonded to glass following an air plasma (PDC-32G Harrick) treatment. Tubing adapters are used to connect the inlets of the device to syringe pumps and to collect solution from the outlets. An optical micrograph of the device is shown in Fig. 1(b). To prevent non-specific cell adhesion to the microfluidic channel walls, the device was coated with bovine serum albumin (Sigma Aldrich) at a concentration of 10 mg per ml and incubated at 4 °C overnight.

### Cell Preparation

Jurkat cells (CRL-1990) were purchased from ATCC. The cells were cultured and maintained in RPMI-1640 medium (Sigma) with the addition of 10% FBS. All cells were incubated at 37 °C with 5% CO_2_. Cells were expanded to 80% confluency in cell culture flasks over two days. To prepare the nonviable cell samples, the cells were heated at 60 °C in a water bath for approximately 2 hours. Heat treatment was chosen over ethanol or chemical treatments to minimize other biomechanical effects such as changes to cell shape. Discarded and deidentified samples of cryopreserved cord blood originally collected from Emory University Hospital under an institutional review board (IRB) approved study for laboratory research on discarded clinical samples and all methods were performed in accordance with the relevant guidelines and regulations. Emory University Hospital obtained the samples from the National Marrow Donor Program (consent was obtained from all participants). The cord blood samples had been previously frozen in media containing 10% v/v DMSO and stored in the vapor phase of liquid nitrogen. The blood was thawed using a warm water bath for 10 minutes b﻿efore e﻿xperiments. For stiffness measurements of nonviable nucleated cord blood cells, heat treatment was applied as described for the Jurkat cells. To avoid clogging in the microfluidics, the blood was diluted 10 times using phosphate buffer saline (PBS) and immediately processed with microfluidics. The viability of unseparated or separated samples of cells was tested by staining with 2 µM ethidium homodimer-1 (EthD-1) (Molecular Probes Inc.) solution^44, 45^. EthD-1 shows strong fluorescence at 635 nm when bound to dead cells. EthD-1 is generally membrane impermeable but capable of only entering damaged or dead cells with compromised cellular membranes. Again, for the identification of caspase-3/7 activity, cells were labeled with 2 µmol/L Cell Event Caspase-3/7 green flow cytometry assay kit (Invitrogen) for 45 minutes at 37 °C and analyzed in flow cytometer. Flow cytometry analysis (Accuri C6, BD) was performed to determine the frequencies of stained non-viable cells and non-stained viable cells.

### Experimental Setup

Jurkat cells were suspended in a phosphate buffered saline solution at a concentration of approximately 1 million cells/ml and infused into the microfluidic device using a syringe pump (PHD 2000, Harvard Apparatus) at specified flow rates. Diluted cord blood was infused into the device at a flow rate of 1.8 ml﻿/hour. Device flow was formed by three inlet streams including two sheath streams which provided hydrodynamic focusing to the third stream containing the cells. The cell trajectories were observed by mounting the microfluidic chip on an inverted bright-field microscope (Eclipse Ti, Nikon) and recorded by high-speed camera (Phantom v7.3, Vision Research) at a frame rate of 2000 frames per second^22^. The high-speed videos were analyzed in *ImageJ* to obtain cell trajectories. The stiffness of live and dead cells was measured using atomic force microscopy (AFM, MFP-3D, Asylum Research). To improve cell stability during AFM measurement, a monolayer of poly-L-lysine (MW 300 kDa, Sigma Aldrich) was applied to gently attach cells to the glass substrate. Beaded silicon nitride cantilevers (spring constant 37.1 pN per nm) were used to indent the center of the cells at a rate of 1.5 μm per second. Sufficient force was applied to achieve at least 5 μm deformation, which is in close comparison with microfluidic compression. Cell Young’s modulus values were calculated from the force-indentation curves by fit to a Hertzian model to compute an average Young’s modulus^46^. For measurement of the stiffness of nucleated cord blood cells, the same procedure was followed except a monolayer of Cell-Tak (BD Biosciences) was used instead of poly-L-lysine to gently attach cells to the glass substrate. One-way analysis of variance (ANOVA) was performed between Young’s modulus of live and dead cells to determine statistical significance. High-resolution optical images were recorded for each cell during AFM measurements to determine cell diameter using *ImageJ*.

### Determination of Diagnostic Odds Ratio

To evaluate the differences in stiffness of live and dead cells as a biomarker, a sensitivity analysis was employed^47^. A confusion matrix was used to divide cells based on condition (positive = live, negative = dead) and test outcome, resulting in frequencies of true positives, false positives, false negatives, and true negatives (TP, FP, FN, and TN, respectively). First, cellular Young’s modulus was considered as the test (soft = test positive, stiff = test negative), and receiver operator characteristic (ROC) curves were plotted by determining the true and false positive rates over the full range of Young’s modulus threshold values. True and false positive rates were calculated using the equations (1) and (2), respectively,

1 $${\rm{True}}\,{\rm{Positive}}\,{\rm{Rate}}=\frac{{\sum }^{}TruePositive}{{\sum }^{}ConditionPositive}$$

2 $${\rm{False}}\,{\rm{Positive}}\,{\rm{Rate}}=\frac{{\sum }^{}FalsePositive}{{\sum }^{}ConditionNegative}$$

The 95% confidence intervals of the receiver operating curves were calculated by bootstrapping using a custom MATLAB code.

The diagnostic odds ratio (DOR) was chosen as a prevalence-independent summary statistic of the ROC curve. The DOR was defined from the frequency of elements in the confusion matrix as DOR = (TP/FP)/(FN/TN). The DOR gives equal weight to false positives and false negatives. The confidence interval bounds of the DOR were calculated by equation (3),

3 $${\rm{DOR}}\pm {\rm{CI}}={{\rm{e}}}^{\mathrm{ln}({\rm{DOR}})\pm {\rm{a}}\sqrt{\frac{1}{{\rm{TP}}}+\frac{1}{{\rm{TN}}}+\frac{1}{{\rm{FP}}}+\frac{1}{{\rm{FN}}}}}$$

where *a* is the inverse of the standard normal cumulative distribution evaluated at $1-\frac{1-{\rm{CI}}}{2}$ and *CI* is the confidence interval^48^.

To calculate the DOR using Young’s modulus as the test condition, AFM data were used to define the frequency of elements in the confusion matrix, where live cells were taken as condition positive and dead cells were taken as condition negative^47^. For the full range of Young’s modulus threshold values, cells with Young’s moduli below the threshold were considered as test positive and cells with Young’s moduli above the threshold were considered as test negative. To calculate the DOR for microfluidic sorting, flow cytometry data were used to define the frequency of elements in the confusion matrix, where live cells were taken as condition positive and dead cells were taken as condition negative. To calculate the DOR for a specific outlet, cells collected from that outlet were considered as test positive and cells collected from all other outlets were considered as test negative.

### Ethics Statements

In this study discarded and deidentified samples of cryopreserved cord blood originally collected from Emory University Hospital under an institutional review board (IRB) approved study for laboratory research on discarded clinical samples and all methods were performed in accordance with the relevant guidelines and regulations. Emory University Hospital obtained the samples from the National Marrow Donor Program (consent was obtained from all participants). Thus, human subjects research was not conducted here.

## Results and Discussion

### Characterization of Live and Dead Cells

**Figure 2:**
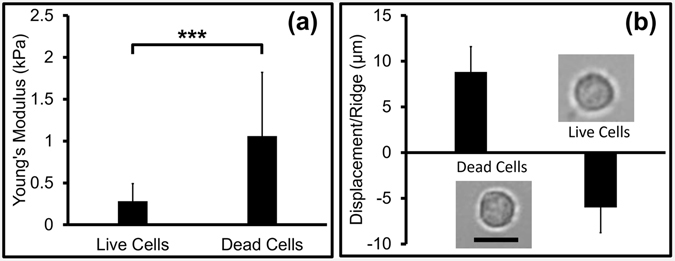
Measurements for live and dead cells (**a**) Young’s modulus of live and dead cells obtained from AFM measurement (***p-value < 0.0001) (*N* = 30); (**b**) average displacement of dead and live cells per ridge in the microfluidic device (*N* = 50); optical micrographs of live and dead cells are shown here (scale bar is 20 µm).

Viable and nonviable heat-treated cells were verified with EthD-1 assay and counted with flow cytometry to be greater than 98% pure (Fig. S1). AFM analysis was conducted on both live and dead cell populations and the average Young’s modulus of live cells was found to be 0.28 ± 0.21 kPa and dead cells 1.06 ± 0.71 kPa (*p*-value < 0.001), shown in Fig. 2(a). The exact mechanisms causing increased stiffness of dead cells are not yet identified, but several hypotheses are suggested^27, 29, 38, 39^. The elasticity of a cell is related to the intrinsic properties of cell membrane and components of the cytoskeleton and cell death may lead to dynamic changes in the actin cytoskeleton of the cells, reduction in cytoplasmic constituents, and cross-linking of the membrane and cytoskeletal structures ultimately leading to increase of the cell stiffness^38, 39, 49^. While some reports observe a change in cell size during apoptosis and necrosis^27, 39, 49^, we did not observe statistically significant size difference in Jurkat cells using the heat-treatment approach. The average diameter of live and dead Jurkat cells was 15.34 ± 2.11 µm and 14.97 ± 2.01 µm, respectively (*N* = 60, *p*-value 0.154). The representative micrographs of live and dead cells are shown in Fig. 2(b).

Live and dead cells were separately flowed through the device at various flow rates to observe their trajectories. Video microscopy showed that the live cells moved perpendicular to the ridges consistent with softness and resulted in a net negative transverse displacement with respect to the direction of fluid flow of −6.01 ± 2.76 µm/ridge (Fig. 2(b)). Dead cells moved in the direction parallel to the ridges consistent with higher stiffness, resulting in a net positive transverse displacement with respect to the direction of fluid flow of 8.84 ± 3.14 µm/ridge (Fig. 2(b)).

The mechanism of cell separation has been investigated previously as a balance between hydrodynamic drag force and elastic force due to cell compression^24^. Since live and dead cells have different stiffnesses, they experience distinct elastic forces as they pass through ridges, but similar hydrodynamic forces. Softer live cells therefore experience a weak elastic force and are directed to the negative transverse direction due to ridge-generated circulatory flow in the microchannel which causes the fluid near bottom surface of the channel to move in the negative transverse direction^42, 50^. On the other hand, stiffer dead cells are translated by a strong elastic force along diagonal ridges towards the stiffer outlet. Consequently, live and dead cells migrate to opposite sides of the ridged microchannel and separated according to their mechanical stiffness^24^.

### Cell Separation in the Microfluidic Device

To study the sorting of live and dead cells, we generated mixtures of live and dead cells at a specified ratio of 1:1 and evaluated the outlets for live and dead cells using flow cytometry. First, the impact of flow rate on trajectories of live and dead cells was studied to optimize the throughput and cell separation. Fluid flow rate was found to impact the ratio of elastic force to hydrodynamic force imposed on cells^22, 24, 43^ and thus the cell trajectory differences. At the tested cell strain of 40%, a higher flow rate (>0.05 ml/min) resulted in both live and dead cells being directed to the bottom/softer outlet and resulted in poor separation. On the other hand, a very low flow rate resulted in occlusion of the channel. The enrichment factor of live and dead cells at all the flow rates are shown in Table 1. At a flow rate of 0.05 ml/min, the average enrichment of live and dead cells was 6.19 and 5.81, respectively. In this case, average flow velocity was 1.2 mm/s and the device processed approximately 830 cells/second. At a lower flow rate of 0.03 ml/min, improved enrichment of live and dead cells (10.08 and 8.65, respectively) was observed. At this flow rate the average flow velocity of cell solution was 0.72 mm/s and the device processed approximately 500 cells/second. The enrichment did not increase at a flow rate of 0.01 ml/min and therefore the remaining experiments were performed at a flow rate of 0.03 ml/min.

**Table 1:** Enrichment factor of live and dead cells at different flow rates.

Flow rate (ml/min)	Enrichment of live cells	Enrichment of dead cells
0.01	8.85	8.09
0.03	10.08	8.65
0.05	6.19	5.81

**Figure 3:**
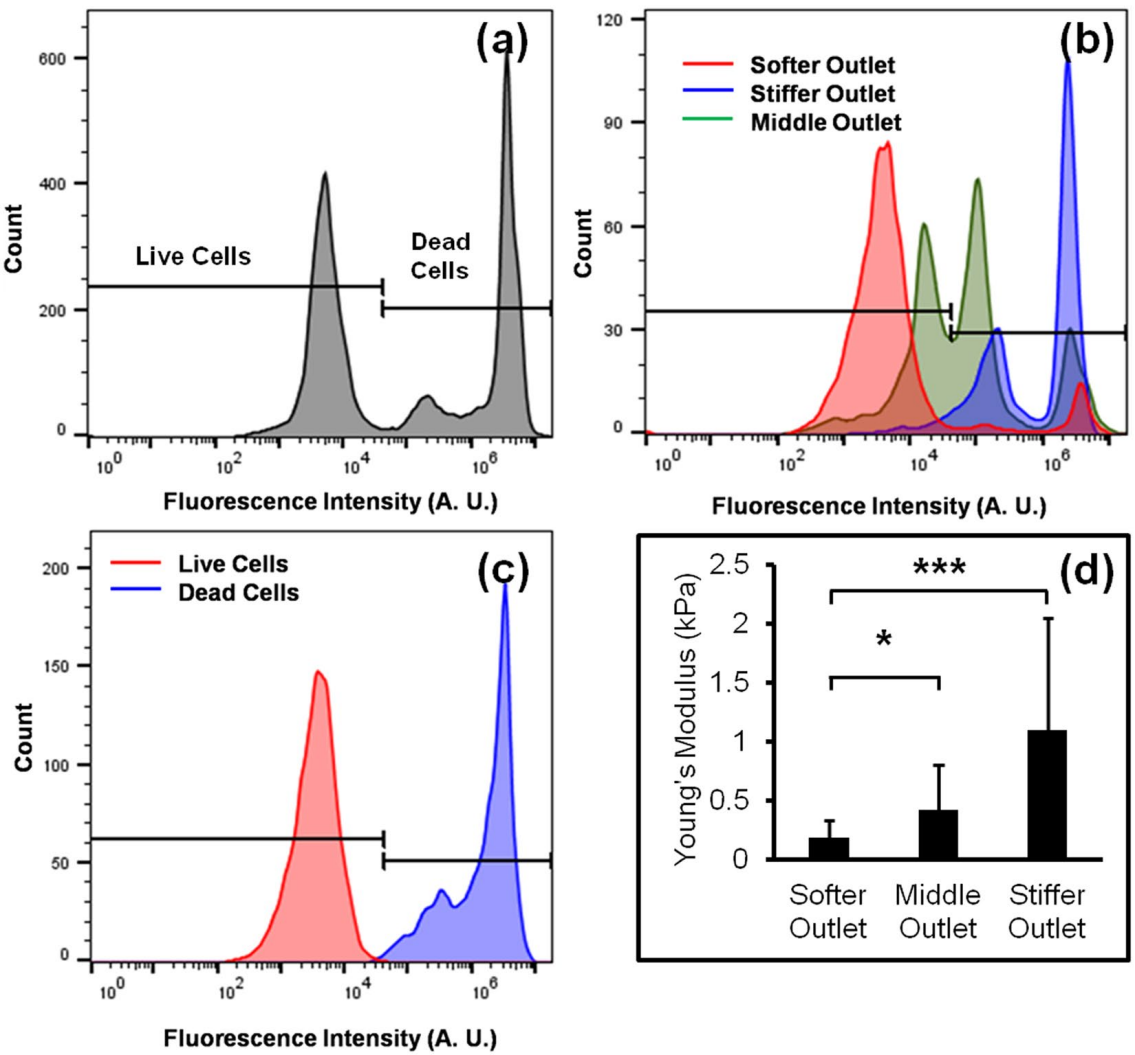
Flow cytometry analysis of the EthD-1 stained cells (**a**) cells in inlet; (**b**) cells collected from three different outlets of the device. The number of live and dead cells was calculated by comparing the results with control experiments; (**c**) flow cytometry data of control live and dead cells stained with Ethd-1 which is used to determine the threshold level to distinguish live and dead cells; (**d**) average Young’s modulus of sorted cells collected from three outlets (*N* = 20, *p-value < 0.01, and ***p-value < 0.001).

Flow cytometry data for cell populations at the inlet and outlets of the device at 0.03 ml/min flow rate are shown in Fig. 3(a and b), respectively. The red, green and blue lines represent the softer, middle and stiffer outlets, respectively. To determine the concentration of live and dead cells, flow cytometry data was compared with the control of no microfluidic processing, shown in Fig. 3(c). The fluorescence intensity of dead cells was higher compared to live cells, as EthD-1 is permeable to dead cells but impermeable to live cells^11^. The concentration of live and dead cells was higher in the softer and stiffer outlets, respectively as shown in Fig. 3(b). The middle outlet was populated with both cell types due to the overlap of biophysical properties between the cell types. Activity of t﻿h﻿e caspase-3/7 gene was also analyzed for the sorted cells using flow cytometer. It was found that the soft outlet contained over 87% cells not labeled by the apoptosis label (shown in Fig. S2), consistent with our understanding of live and dead sorting. An enrichment factor at each flow rate was calculated from the ratio of live and dead cells from each outlet and the inlet, as described by the following equation:

4 $${\rm{Enrichment}}\,{\rm{Factor}}=\frac{Ratio\,of\,live\,and\,dead\,cells\,in\,softer\,outlet}{Ratio\,of\,live\,and\,dead\,cells\,in\,inlet}$$

To validate that the sorting mechanism was dependent upon mechanical stiffness, AFM analysis was performed on the sorted cells. The Young’s modulus of the softer outlet was significantly lower compared to stiffer outlet (*p*-value < 0.01), as shown in Fig. 3(d).

**Figure 4:**
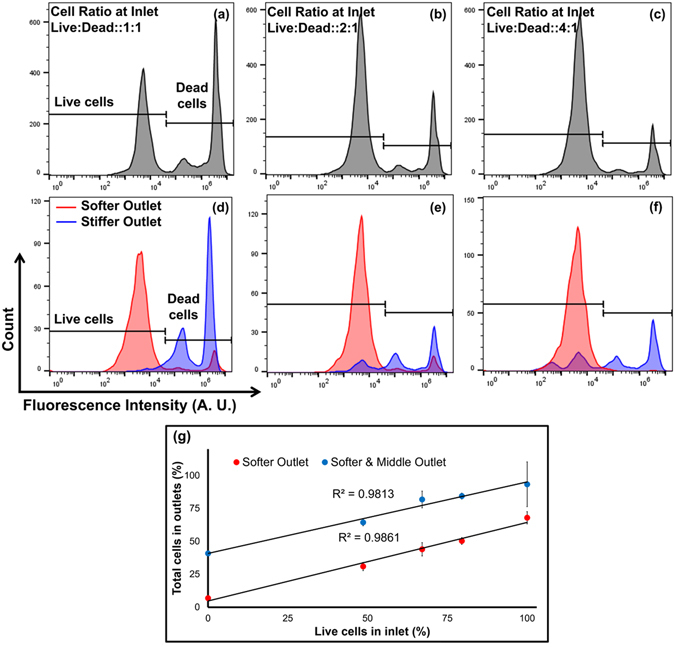
The flow cytometry results for different ratios of cells in the inlet; approximate ratio of live and dead cells in inlet (**a**) 1:1; (**b**) 2:1; (**c**) 4:1; the data obtained from softer and stiffer outlets for the ratio of live and dead cells a﻿t inlet (**d**) 1:1; (**e**) 2:1; (**f**) 4:1; (**g**) percentage of live cells in softer outlet for different percentage of live cells in inlet. For (**a**–**f**), *x* and *y*-axis are representing fluorescence intensity and cell counts, respectively.

In more realistic cell assay scenarios, the number of dead cells would be expected to be lower than the number of live cells. Therefore, we tested enrichment for a range of live to dead cell ratios. The enrichment factor and resulting purity of sorted cells was also measured for initial live to dead cell ratios of 2:1 and 4:1 and plotted in Fig. 4 at a flow rate of 0.03 ml/min. The purity of the live cells was calculated from the ratio of live cells and total number of cells in the softer outlet. By increasing the ratio from 1:1 to 4:1, the purity of live cells was elevated from 88.67% to 98.1% in the softer outlet. The enrichment factor was also increased to some extent for the higher ratio of live and dead cells provided in Table 2. Flow cytometry data obtained from the cell mixture at the inlet, softer, and stiffer outlets are shown in Fig. 4(a–f). The outlet data showed an enhanced concentration of live cells at the softer outlets and dead cells at the stiffer outlets. Flow cytometry results showed a very small percentage of dead cells in softer outlets (Fig. 4(e,f)). We hypothesize that the reduced numbers of stiff, dead cells improved the flow of all cells and reduced interference of the natural trajectories within the device and led to higher overall purity. The overall high purity in all cases indicated that only a small percentage of live cells (less than 8%) were directed to the stiffer outlet. It was found that the percentage of cells at the softer outlet was linearly proportional to the percentage of live cells at the inlet (Fig. 4(g)), validating the sensitivity of the device to viable cells.

**Table 2:** Enrichment of live and dead cells at different ratios of cells a﻿t the inlet.

Live:Dead (at inlet)	Enrichment of Live Cells	Purity of Live Cells	Enrichment of Dead Cells
1:1	10.08	88.67%	8.65
2:1	11.04	95.63%	8.11
4:1	13.47	98.10%	7.27

**Figure 5:**
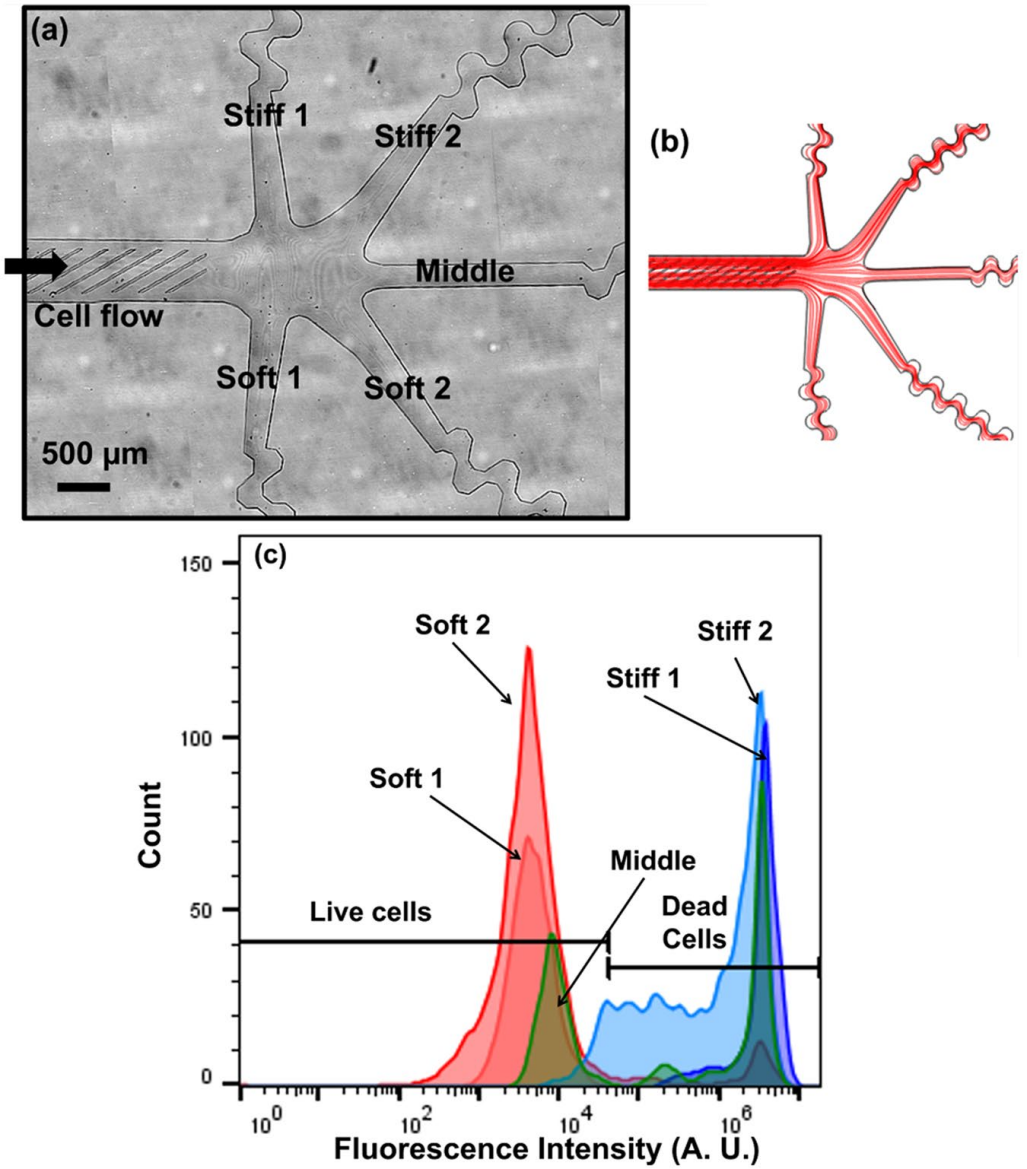
Five outlets device. (**a**) Schematic diagram of the five outlets device; (**b**) simulated streamline of fluid flow in the device (**c**) flow cytometry results obtained from different outlets of the device.

In the three outlet device, the majority of the live cells were collected at the softer outlet (~57%) but a substantial percentage of live cells were also directed to the middle outlet as well (~35%). If a balance between live cell recovery (sensitivity) and live cell enrichment (specificity) is desired, then the current chip design would collect both the soft and middle outlet, resulting in a live cell collection efficiency of 92% and an enrichment of only 3-fold. To test whether increasing the fractionation of the sorted populations could result in both improved sensitivity and specificity, we tested a five-outlet sorting device^51^. The optical micrograph of the device is shown in Fig. 5(a) and the simulated streamline in the device is shown in Fig. 5(b). This device was designed to have two soft (*soft 1* and *soft 2*), one middle, and two stiff (*stiff 1* and *stiff 2*) outlets.

The experiment was performed with a starting ratio of 1:1 for live and dead cells at a flow rate of 0.03 ml/min. The flow cytometry results obtained from different outlets are shown in Fig. 5(c). The enrichment for live cells in *soft 1* outlet was 185-fold leading to purity of over 99%. However, this high enrichment was associated with recovery of only 28.8% of live cells in a single outlet. Combining the cells in both soft outlets resulted in recovery of 84.0% live cells with 94.9% purity and 17.3 times enrichment shown in Table 3. The recovery of the live cells can be increased to 95.6% with a purity of 82.4% by including cells from middle outlet.

**Table 3:** Purity and enrichment obtained from 5 outlet﻿s device.

Outlets	Purity (%)	Enrichment	Recovery rate for live cells (%)
Soft 1	99.50	185.17	28.82
Soft 2	92.10	10.85	53.93
Soft 1 + Soft 2	94.55	16.14	82.74
Soft 1 + Soft-2 + Middle	82.36	4.34	95.11

We verified that the periodic compression by the ridges did not cause cell death at the strains studied (Fig. S3). To confirm that sorted cells could be further processed post-sort, we resuspended the viable cells in cell culture medium and continued cell growth under culture conditions for seven days. The cell concentration was calculated at different time points using a hemocytometer, shown in Fig. S4. As control, we also cultured the cells from the inlet before they had passed through the device. The sorted live cells grew substantially over this time period at a rate (0.22 ± 0.04 day^−1^) higher than the control samples (0.12 ± 0.01 day^−1^).

### Diagnostic Odds Ratio

**Figure 6:**
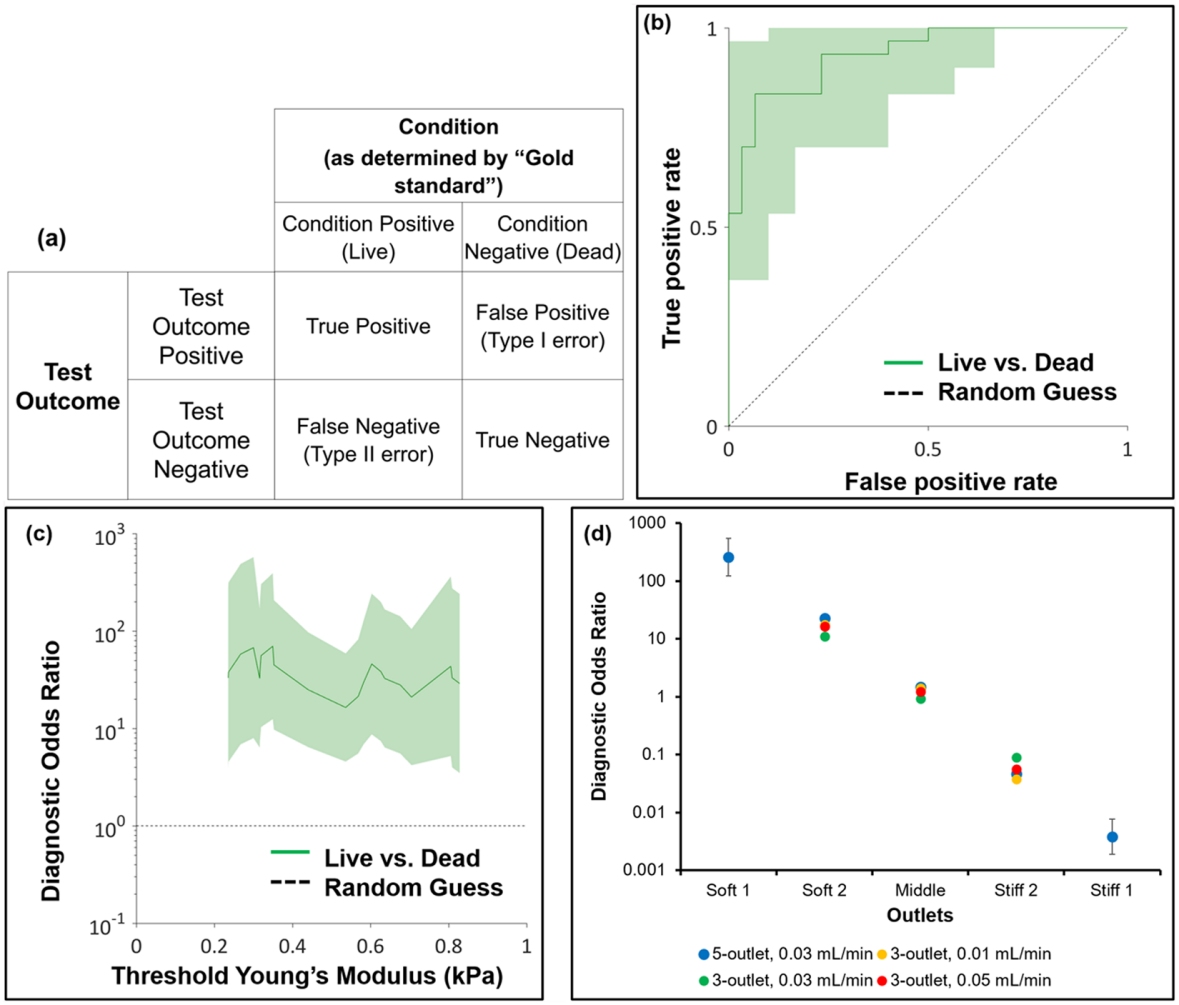
(**a**) Confusion matrix to compare the known condition to the test outcome; (**b**) ROC for Young’s moduli of live and dead cells; (**c**) DOR for Young’s moduli of live and dead cells; (**d**) DOR for sorted outlets at different outlets for both (**b**,**c**) solid green live is for live vs. dead cells, dashed black line is for random guess, and shaded green area represents the 95% confidence interval, for (**d**) error bars indicate the 95% confidence interval.

The confusion matrix (Fig. 6(a)) was used to divide cells based on conditions. For a perfect test, the ROC curve approaches to the top-left corner of the true positive rate vs. false positive rate plot where both false positive (FP) and false negative (FN) are zero^47^. Young’s modulus threshold values within the 95% confidence interval outperformed a random test because the Young’s modulus curve has a higher true positive rate and a lower false positive rate than the random guess curve for all points, as shown in Fig. 6(b). Here, the green line represents live versus dead classification, the dashed black line indicates a random guess classification and the shaded green area represents the 95% confidence interval. The area under the ROC curve (AUC) also indicates the utility of a test, where a perfect test has an AUC of 1^47^. In case of Young’s modulus, the AUC value was 0.933 (95% confidence interval: 0.798–0.997). Next, the DORs for Young’s modulus of the cells were calculated shown in Fig. 6(c). Again threshold Young’s moduli within the 95% confidence interval outperform a random test (DOR = 1) for all points. The DOR reached a maximum of 70 at a threshold Young’s modulus of 350 Pa. Next, the DOR was determined for sorted cells from different outlets shown in Fig. 6(d). For the 3-outlet devices under all flow conditions considered, the DOR showed a maximum of 15 for the softer outlet and a minimum of 0.05 for the stiffer outlet (DOR < 1 indicates enrichment of dead cells). By increasing the number of outlets with the 5-outlet device, the DOR was further improved to 136 in the *soft1* outlet suggesting that the odds of a cell reaching this outlet is 136 times higher for live cells than for dead cells.

### Enriching the Viability of Cord Blood Cells

**Figure 7:**
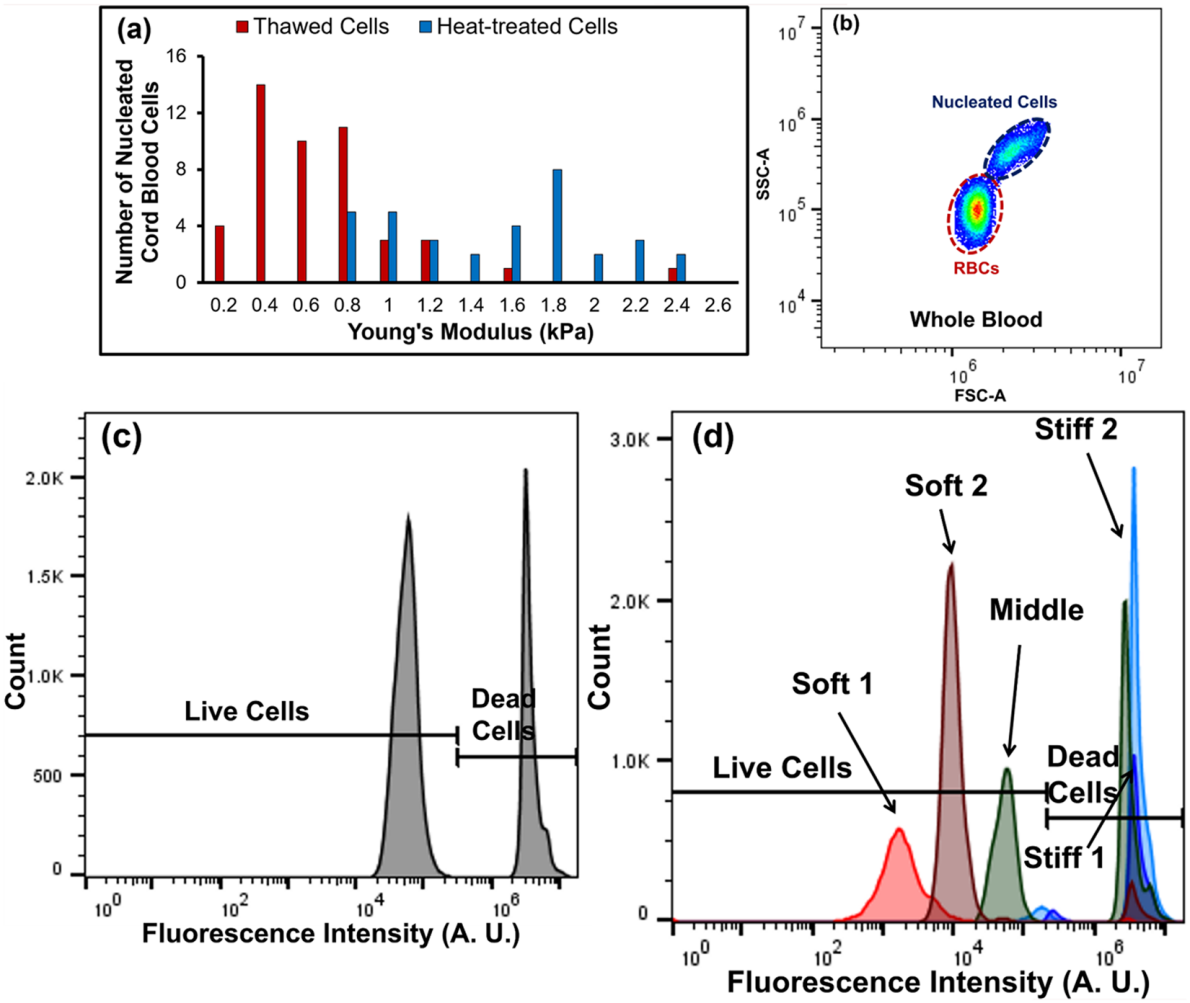
AFM data and flow cytometry results obtained from cord blood (**a**) distribution of Young’s modulus for nucleated cord blood cells (*N* > *30*); (**b**) scatter plot of cord blood to distinguish nucleated cells from red blood cells; (**c**) ﻿EthD﻿-1 staining of live and dead nucleated cells in the cord blood; (**d**) viability of nucleated cells collected from different outlets after microfluidic sorting.

To validate the utility of viability sorting in a therapeutic context, microfluidic sorting was used to improve the purity of viable cells from samples of thawed cord blood that were rich in hematopoietic stem cells. The Young’s moduli of nucleated cells from thawed cord blood samples and heat-treated, nonviable samples were measured separately using AFM. Figure 7(a) shows the stiffness histogram of the samples. We note that the heat-treated cells sample is uniformly stiffer than the thawed sample, and that there is a region of overlap, consistent with the existence of a nonviable subpopulation within the thawed sample. The average Young’s modulus for the nucleated cord blood cells that were thawed was 0.57 ± 0.37 kPa and after heat treatment this value increased to 1.44 ± 0.49 kPa. After this characterization, the microfluidic device was implemented to sort the thawed cord blood sample for viability. Frozen cord blood was thawed and diluted 10 times using cell buffer and the cells were processed by the device. The thawed cells sent to the inlet and sorted cells collected from all five outlets were stained with EthD-1 and flow cytometry was performed to calculate the percentage of viable nucleated cells. Flow cytometry was used to identify nucleated cells and red blood cells, shown in Fig. 7(b). The nucleated cells showed a distinctive profile in the forward and side scatter plot due to their difference in size and granularity which was gated for the stain analysis. The percentage of viable nucleated cells in the blood was determined from fluorescence intensity and found to be 65.2% post-thaw (Fig. 7(c)). Flow cytometry results from the outlets are shown in Fig. 7(d) and the viability data are given in Table 4. The combination of both soft outlets recovered 73.3% of the live cells with 94.8% purity and 9.77-fold enrichment. The recovery of the live cells can be increased to 97.5% with a purity of 76.1% by including cells from the middle outlet.

**Table 4:** Purity and enrichment obtained for cord blood.

Outlets	Purity (%)	Enrichment	Recovery (%)
Soft 1	98.4	32.83	27.48
Soft 2	92.8	6.88	45.84
Soft 1 + Soft 2	94.8	9.77	73.32
Soft 1 + Soft 2 + Middle	76.1	1.70	97.51

## Conclusion

This study showed that stiffness can be an effective biomarker by which to sort viable cells from nonviable cells. The unique repeated skew ridge microchannel design is effective to sort live and dead cells based upon differences in cell stiffness with high purity and high collection efficiency. We observed enrichment over 185-fold for live cells with purity of over 99% from the mixture of live and dead cells. Sorting viable cells with high purity and recovery was achieved with no labeling of the cells, which offers significant practical advantages over the existing label-based sorting methods. This simple device can be implemented within the laboratory much like a filter or directly integrated in a microfluidic lab-on-a-chip to purify cell samples to higher viability to improve the quality of downstream analyses. With the emerging importance of cell manufacturing, the microfluidics approach may be effective to continuously process cells to improve the quality of the product and better implement proficiency testing to obtain consistent potency and safe cell therapies.

## Electronic supplementary material

Movie S2

Movie S1

Supplementary

## References

[1] Tennant JR (1964). Evaluation of the trypan blue technique for determination of cell viability. Transplantation.

[2] Nagy A, Rossant J, Nagy R, Abramow-Newerly W, Roder JC (1993). Derivation of completely cell culture-derived mice from early-passage embryonic stem cells. Proceedings of the National Academy of Sciences.

[3] Ishiyama M (1996). A combined assay of cell viability and *in vitro* cytotoxicity with a highly water-soluble tetrazolium salt, neutral red and crystal violet. Biological and Pharmaceutical Bulletin.

[4] Akel S (2014). Current thawing and infusion practice of cryopreserved cord blood: the impact on graft quality, recipient safety, and transplantation outcomes. Transfusion.

[5] Joza N (2001). Essential role of the mitochondrial apoptosis-inducing factor in programmed cell death. Nature.

[6] Lee S (2008). Post-thaw viable CD34+ cell count is a valuable predictor of haematopoietic stem cell engraftment in autologous peripheral blood stem cell transplantation. Vox Sanguinis.

[7] Allan DS (2002). Number of viable CD34 (+) cells reinfused predicts engraftment in autologous hematopoietic stem cell transplantation. Bone Marrow Transplantation.

[8] Yang H (2005). Association of post-thaw viable CD34 & plus; cells and CFU-GM with time to hematopoietic engraftment. Bone Marrow Transplantation.

[9] Patel S (2012). Microfluidic separation of live and dead yeast cells using reservoir-based dielectrophoresis. Biomicrofluidics.

[10] Jen C-P, Chen T-W (2009). Selective trapping of live and dead mammalian cells using insulator-based dielectrophoresis within open-top microstructures. Biomedical Microdevices.

[11] Shafiee H, Sano MB, Henslee EA, Caldwell JL, Davalos RV (2010). Selective isolation of live/dead cells using contactless dielectrophoresis (cDEP). Lab on a Chip.

[12] Docoslis A, Kalogerakis N, Behie LA, Kaler KVIS (1996). A novel dielectrophoresis-based device for the selective retention of viable cells in cell culture media. Biotechnology and Bioengineering.

[13] Achieving large-scale, cost-effective, reproducible manufacturing of high-quality cells: A technology roadmap to 2025. Consortium, N. C. M. (2016).

[14] Hunsberger J (2015). Manufacturing road map for tissue engineering and regenerative medicine technologies. Stem Cells Transl Med.

[15] Minimally manipulated, unrelated allogeneic placental/umbilical cord blood intended for hematopoietic reconstitution for specified indications. *US Department of Health and Human Services Food and Drug Administration Center for Biologics Evaluation and Research* (2009).

[16] Fenske TS (2013). Autologous or reduced-intensity conditioning allogeneic hematopoietic cell transplantation for chemotherapy-sensitive mantle-cell lymphoma: analysis of transplantation timing and modality. Journal of Clinical Oncology.

[17] Wirk B (2014). Outcomes of hematopoietic cell transplantation for diffuse large B cell lymphoma transformed from follicular lymphoma. Biology of Blood and Marrow Transplantation.

[18] Tanaka T (2003). Hypoxia-induced apoptosis in cultured glomerular endothelial cells: involvement of mitochondrial pathways. Kidney International.

[19] Nakamura M, Esumi H, Jin L, Mitsuya H, Hata H (2008). Induction of necrosis in human myeloma cells by kigamicin. Anticancer Research.

[20] Gregory, C. Inventors; Grampian Biopartners Limited, assignee. Method for separating viable cells, apoptotic and dead cells. United States patent US 8470978 B2. Jun 25 (2013).

[21] Penberthy KK, Ravichandran KS (2016). Apoptotic cell recognition receptors and scavenger receptors. Immunological Reviews.

[22] Wang G (2015). Microfluidic cellular enrichment and separation through differences in viscoelastic deformation. Lab on a Chip.

[23] Wang G (2015). Cellular enrichment through microfluidic fractionation based on cell biomechanical properties. Microfluidics and Nanofluidics.

[24] Wang G (2013). Stiffness dependent separation of cells in a microfluidic device. PLoS One.

[25] Islam M (2015). Effects of nanotexture on electrical profiling of single tumor cell and detection of cancer from blood in microfluidic channels. Scientific Reports.

[26] Islam M, Asghar W, Young-tae K, Iqbal SM (2014). Cell elasticity-based microfluidic label-free isolation of metastatic tumor cells. British Journal of Medicine and Medical Research.

[27] Hessler JA (2005). Atomic force microscopy study of early morphological changes during apoptosis. Langmuir.

[28] Meng F-y, Chen Z-s, Han M, Hu X-p, Zhou P (2010). An improved purification approach with high cell viability and low cell loss for cryopreserved hepatocytes. Cryobiology.

[29] Cerf A, Cau J-C, Vieu C, Dague E (2009). Nanomechanical properties of dead or alive single-patterned bacteria. Langmuir.

[30] Rafaï S, Jibuti L, Peyla P (2010). Effective viscosity of microswimmer suspensions. Physical Review Letters.

[31] Frame KK, Hu W-S (1990). Cell volume measurement as an estimation of mammalian cell biomass. Biotechnology and Bioengineering.

[32] Lei U, Sun P-H, Pethig R (2011). Refinement of the theory for extracting cell dielectric properties from dielectrophoresis and electrorotation experiments. Biomicrofluidics.

[33] Vahey MD, Voldman J (2008). An equilibrium method for continuous-flow cell sorting using dielectrophoresis. Analytical Chemistry.

[34] Li H, Bashir R (2002). Dielectrophoretic separation and manipulation of live and heat-treated cells of Listeria on microfabricated devices with interdigitated electrodes. Sensors and Actuators B: Chemical.

[35] Lewpiriyawong N, Kandaswamy K, Yang C, Ivanov V, Stocker R (2011). Microfluidic characterization and continuous separation of cells and particles using conducting poly (dimethyl siloxane) electrode induced alternating current-dielectrophoresis. Analytical Chemistry.

[36] Doh I, Cho Y-H (2005). A continuous cell separation chip using hydrodynamic dielectrophoresis (DEP) process. Sensors and Actuators A: Physical.

[37] Jen C-P, Chen W-F (2011). An insulator-based dielectrophoretic microdevice for the simultaneous filtration and focusing of biological cells. Biomicrofluidics.

[38] Lulevich V, Zink T, Chen H-Y, Liu F-T, Liu G-y (2006). Cell mechanics using atomic force microscopy-based single-cell compression. Langmuir.

[39] Lam WA, Rosenbluth MJ, Fletcher DA (2007). Chemotherapy exposure increases leukemia cell stiffness. Blood.

[40] Choi S, Song S, Choi C, Park J-K (2007). Continuous blood cell separation by hydrophoretic filtration. Lab on a Chip.

[41] Choi S, Karp JM, Karnik R (2012). Cell sorting by deterministic cell rolling. Lab on a Chip.

[42] Mao W, Alexeev A (2011). Hydrodynamic sorting of microparticles by size in ridged microchannels. Physics of Fluids.

[43] Bongiorno T (2014). Mechanical stiffness as an improved single-cell indicator of osteoblastic human mesenchymal stem cell differentiation. Journal of Biomechanics.

[44] Barron JA, Ringeisen BR, Kim H, Spargo BJ, Chrisey DB (2004). Application of laser printing to mammalian cells. Thin Solid Films.

[45] Zhou H-m (2015). *In Vitro* cytotoxicity of calcium silicate-containing endodontic sealers. Journal of Endodontics.

[46] Xu W (2012). Cell stiffness is a biomarker of the metastatic potential of ovarian cancer cells. PloS one.

[47] Bongiorno T, Chojnowski J, Lauderdale J, Sulchek T (2016). Cellular stiffness as a novel stemness marker in the corneal limbus. Biophysical Journal.

[48] Glas AS, Lijmer JG, Prins MH, Bonsel GJ, Bossuyt PMM (2003). The diagnostic odds ratio: a single indicator of test performance. Journal of Clinical Epidemiology.

[49] Kim KS (2012). AFM-detected apoptotic changes in morphology and biophysical property caused by paclitaxel in Ishikawa and HeLa cells. PloS One.

[50] Arata JP, Alexeev A (2009). Designing microfluidic channel that separates elastic particles upon stiffness. Soft Matter.

[51] Di Carlo D, Edd JF, Irimia D, Tompkins RG, Toner M (2008). Equilibrium separation and filtration of particles using differential inertial focusing. Analytical Chemistry.

