# Supplementary material for: Microfluidic Sorting of Cells by Viability Based on Differences in Cell Stiffness
Source: Sci Rep. 2017 May 17;7:1997. doi: 10.1038/s41598-017-01807-z (PMC5435733; doi:10.1038/s41598-017-01807-z)
Supplement: Supplementary file 3 — Supplementary [file 41598_2017_1807_MOESM3_ESM.pdf]

Microfluidic Sorting of Cells by Viability Based on Differences in Cell Stiffness

Muhymin Islam[a], Hannah Brink[b], Syndey Blanche[b], Caleb DiPrete[b], Tom Bongiorno[a], Nicholas Stone[a], Anna Liu[b], Anisha Philip[c], Gonghao Wang[a], Wilbur Lam[b,c,d], Alexander Alexeev[a], Edmund K. Waller[d], and Todd Sulchek[*ab]

[a]George W. Woodruff School of Mechanical Engineering, Georgia Institute of Technology, 801 Ferst Drive, Atlanta, GA, 30332-0405, USA

[b]Wallace H. Coulter Department of Biomedical Engineering, Georgia Institute of Technology, 313 Ferst Drive, Atlanta, GA, 30332-0535, USA

[c]Department of Pediatrics, Division of Pediatric Hematology/Oncology, Aflac Cancer Center and Blood Disorders Service of Children's Healthcare of Atlanta, Emory University School of Medicine, Atlanta, Georgia 30322

[d]Winship Cancer Institute, Emory School of Medicine, 1365 Clifton NE Rd: Atlanta, GA 30322

*E-mail: todd.sulchek@me.gatech.edu

Supplementary Information

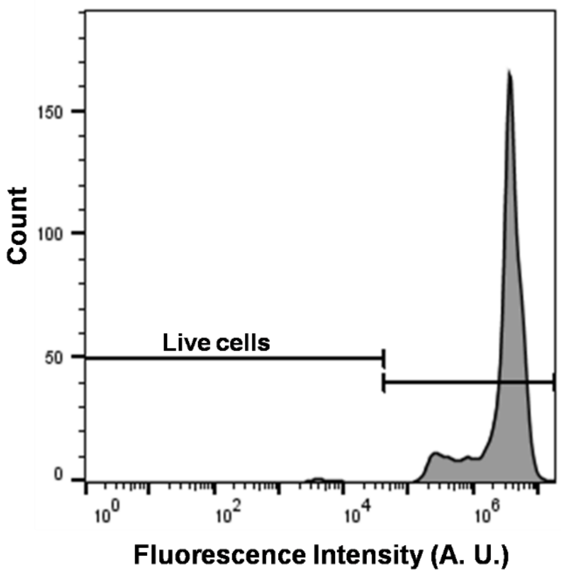

Fig. S1: Dead cells were stained with EthD-1 and flow cytometry results ensured death of more than 98% cells.

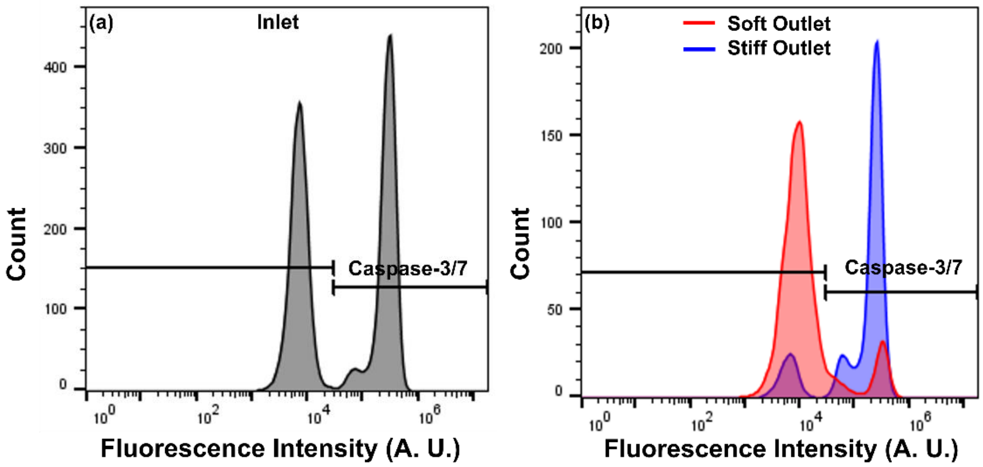

Fig. S2: Activity of Caspase-3/7 gene of the sorted cells. Flow cytometry data from (a) inlet; (b) outlets.

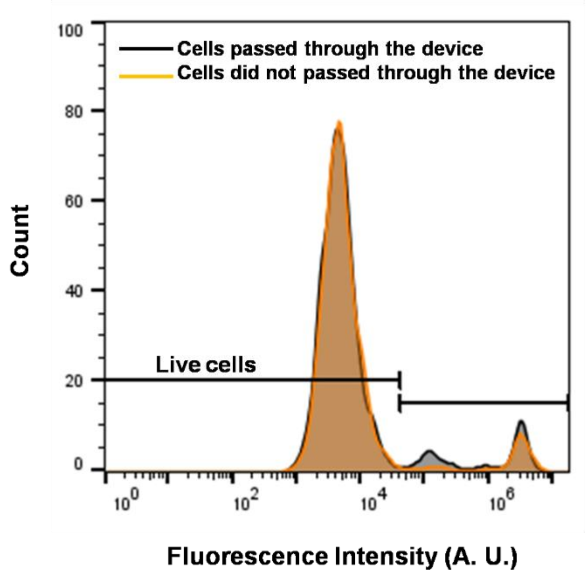

Fig. S3: Flow cytometry analysis data of control experiment showed that the periodic compression of the ridges introduced approximately only 3.2% cell death.

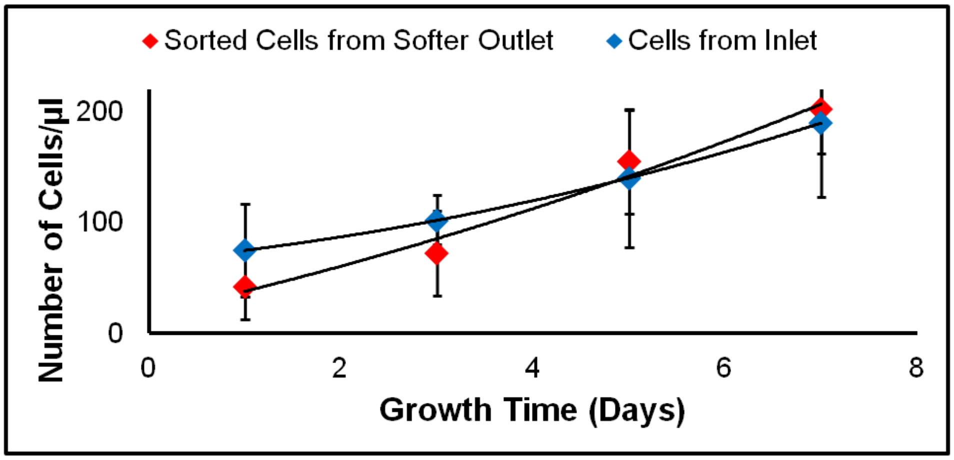

Fig. S4: Growth of sorted live Jurkat cells collected after flow experiment. The control cells were not passed through the device. Processed cells showed a lower initial concentration due to the dilution of the sheath flow.

Movie S1: The movement of live cells in the device towards soft and middle outlets.

Movie S2: The movement of live and dead cells in the device towards the outlets when they were mixed at a ratio of 1:1 in the inlet.

Table S1: Innovation of the study compared to previous work:

**Innovations of the study**	**Current study**	**Earlier studies[1-3]**
Stiffness as a label-free biomarker for cell viability	Established that a variety of methods to induce cell apoptosis and death results in increases of cell stiffness at an early time point. The viability biomarker can be determined through both atomic force microscopy and stiffness sorting	While we have previously shown microfluidics can be used to sort cells by stiffness, these cells were either of different cell line types, for example K562, HL60[1-3], or modified chemically[1].
Stiffness measurement of nucleated cord blood cells	For the first time, we have measured the stiffness of nucleated cord blood cells using atomic force microscopy.	Previously we measured stiffness only for different cell lines[1-3] or for primary cells[4-5] not derived from cord blood.
Purification of cord blood	We have implemented our device for the purification of cord blood by eliminating the stiff, nonviable cells from the sample.	No such application was shown.
5-outlets device	For the first time, we have designed and implemented ridge based microfluidic device with 5-outlets such that the recovery and purity can be chosen based upon which outlets are pooled and collected.	Previously, we have implemented 2-oultets[1]or 3-oultets[3] devices for biomechanical sorting.
Number of ridges	The number of ridges was optimized to 14 for 5-outlets device based upon empirical data that show trajectory divergence cease as cells lose their effective compliance.	The number of ridges used previously was 21 or higher[1-3].
Outlet design	Diameter of the outlets was increased to 3 mm for increased cell collection.	Diameter of the outlets were 1 mm[1-3].

	The expansion region before the outlets and angular displacement of the outlets were redesigned computationally for 5-outlets device to distribute the channel flow uniformly to ensure better separation efficiency for viable and nonviable cells.	The expansion region and angular displacement of the outlets were designed to distribute the channel flow to increase the collection of soft cells[3].
Device resolution and performance	5-outlets device has 1.67 times better resolution compared to 3-outlets device for stiffness based sorting.	3-outlets device has 1.67 times lower resolution compared to 5-outlets device.
	Enrichment up to 185-fold was obtained.	The enrichment was up to 45-fold[3].
	For equal amount of viable and nonviable cells in the inlet, the purity of viable cells was improved to 99.50% in *soft 1* outlet. The recovery rate was also increased to 95% by combining *soft 1*, *soft 2* and middle outlets.	For equal amount of viable and nonviable cells in the inlet, the purity of viable cells was 88.67% with recovery rate of only 57%.
Study of genetic properties	Studied genetic properties (caspase activity) of sorted cells using flow cytometry.	Did not study genetic properties of sorted cells.
Statistical analysis	We have performed a statistical analysis of the accuracy of sorting by determining the diagnostic odds ratio and receiver operator curves to validate that our sorting was based on stiffness.	No such statistical analysis was performed previously.

References:

1. Wang, G. *et al.* Stiffness dependent separation of cells in a microfluidic device. *PLoS One* **8,** e75901 (2013).
2. Wang, G. *et al.* Microfluidic cellular enrichment and separation through differences in viscoelastic deformation. *Lab on a Chip* **15,** 532-540 (2015).
3. Wang, G. *et al.* Cellular enrichment through microfluidic fractionation based on cell biomechanical properties. *Microfluidics and Nanofluidics* **19,** 987-993 (2015).
4. Bongiorno, T. *et al.* Mechanical stiffness as an improved single-cell indicator of osteoblastic human mesenchymal stem cell differentiation. *Journal of Biomechanics* **47,** 2197-2204 (2014).
5. Meredith, F. *et al.* Cellular softening mediates leukocyte demargination and trafficking, thereby increasing clinical blood counts. *Proceedings of the National Academy of Sciences* **13,** 1987-1992 (2016).
